# Unlock the innovation potential of meaning of work: An empirical study of scientific and technological workers in China

**DOI:** 10.3389/fpsyg.2022.870318

**Published:** 2022-07-22

**Authors:** Kexin Liang, Sheng Lin, Jinlan Liu, Yifan Zhu

**Affiliations:** College of Management and Economics, Tianjin University, Tianjin, China

**Keywords:** meaning of work, innovative behavior, organizational innovative climate, scientific and technological workers, achievement motivation

## Abstract

Creativity and innovation have significantly increased in the past years. Amabile and Pratt were the leading proponents of creativity who integrated a dynamic componential model of creativity and innovation in organizations. The present study discusses the concept of innovative behavior within the scientific and technological environment based on the dynamic componential model of creativity and innovation and the Triadic Reciprocal Determinism Theory. The study investigates the mediating effect of achievement motivation and the moderating effect of the organizational innovative climate between the meaning of work and innovative behavior. Meaning of work has a positive impact on innovative behavior based on the structural equation modeling and the results of data collected from the survey of 4,666 scientific and technological workers in China. In addition, achievement motivation plays a partial intermediary role between the meaning of work and innovative behavior. However, innovation within organizational climate plays a negative regulatory role between achievement motivation and innovative behavior. The study finds some existing weaknesses through the Importance-Performance Map Analysis. Lastly, we examine the critical findings and present hypothetical suggestions.

## Introduction

Creative employees with innovative ideas are highly valued by organizations, as various kinds of work are now increasingly knowledge-intensive. Innovative work behaviors often help organizations to enhance competitive market advantages, especially in knowledge-intensive industries (Anderson et al., 2014). Studies confirmed that compulsory compensation and different forms of rewards are effective tools for enhancing employees’ innovative behaviors (Malik and Butt, 2017). Although previous studies explore factors affecting innovative behavior from different perspectives, which include psychological empowerment (Rehman et al., 2019), organizational culture and environment, management support and organizational learning (Walker, 2016), and work engagement (Cheng et al., 2020), these factors do not fully explain the innovative work behavior of Chinese scientific and technological workers.

First, in Chinese modern society, the trend of employees pursuing a sense of meaning in their work is becoming more and more obvious, and many next-generation employees are involved in aspirational thinking and organizational control. Work is more than earning means of livelihood; workers aim to reflect their values and demonstrate their skills. Many studies have argued that experiencing meaning at work helps employees pursue work goals and engage in productive activities (Steger and Dik, 2009; Magni et al., 2018). Specific to this study, scientific and technological workers take innovation as their main work content, and employees who do meaningful work may be more motivated to meet the challenges of innovation. Research shows that experiencing meaningful work may make an impact by making employees feel more likely to benefit the organization as they innovate (Dutton, 2001). In addition, they may be more likely to engage in innovative and creative behavior in the workplace (Grant and Berry, 2011). Also, the dynamic componential model of creativity and innovation, which is the most well-known theory in the field of creativity, adds “meaningful work” as a new factor that affects the creative process. The studies emphasize that meaningful work is not critical to explaining the intrinsic motivation of engaging in creative work and re-participate through the schedule cycle. The theory is also important in explaining the effectiveness of leaders’ innovation-related statements in enhancing employees’ intrinsic motivation (Amabile and Pratt, 2016).

Second, while studies have shown that meaningful work improves employees’ innovative behavior, existing research has rarely explored the specific mechanisms by which it acts on innovative behavior. Studies have shown that people who have experienced meaningful work often feel intrinsically motivated (Amabile and Pratt, 2016). Thus, a positive response to facing challenges and problems in an innovative way may be appealed (Yidong and Xinxin, 2013). Achievement motivation is considered by this institute as a suitable type of motivation. People with high achievement motivation have high motivation to overcome and achieve difficult things. Scholars have shown that in the hospitality management industry, the meaning of work as a dimension of psychological empowerment can improve employee productivity by promoting various motivations for need, including achievement motivation (Amenumey and Lockwood, 2008).

Finally, according to the dynamic componential model of creativity and innovation and Triadic Reciprocal Determinism Theory, organizational innovation climate influences the extent to which employees’ achievement motivation affects innovative behavior. Many previous studies have also confirmed that the organizational innovation climate acts as a moderator of the innovation behavior of employees. Therefore, in order to fully understand the innovative behavior of scientific and technological workers, it is necessary to explore how the organizational innovation climate moderates the impact of achievement motivation on innovative behavior.

In the following sections, we first review the research on the relationship between the meaning of work, achievement motivation, organizational innovation climate, and innovative behavior. Specifically, this study is based on the dynamic componential model of creativity and innovation proposing that the meaning of work affects the innovative behavior of scientific and technological workers. Also, it is based on Triadic Reciprocal Determinism Theory, considering achievement motivation as the mediating variable and the organizational innovation climate as the moderating variable. Based on a literature review, we designed a questionnaire and proposed and validated this hypothesis from a data analysis of 4,666 scientific and technological workers in China. We use Importance-Performance Map Analysis (IPMA) to find some practical discoveries, improve the meaning of work for scientific and technological workers, and enhance their innovative behavior. Finally, this paper concludes with a discussion of the significance of the findings, the limitations of the study, and future research recommendations.

Our research also provides the following contributions. First of all, this paper empirically studies the impact of the meaning of work on innovative behavior that verifies and expands the dynamic componential model of creativity and innovation (Amabile and Pratt, 2016). In previous studies, the relationship between the meaning of work and innovative behavior has not been clarified, and few empirical studies have investigated the relationship between the two. Second, achievement motivation is added as the mediate variable to verify the mechanism of the meaning of work for innovative behavior stimulates the vitality and commitment to work by enhancing the achievement motivation of scientific and technological workers. Finally, this paper emphasizes the difference in the influence of scientific and technological workers’ achievement motivation on innovative behavior in different intensities of organizational innovation climate and expands the Trait activation theory.

## Literature review

### Meaning of work and innovation behavior

In the early research, the meaning of work was regarded as a static feature of work. Scholars generally believed that the meaning of work was the characteristic of the workplace, where employees fulfilled the general work requirements (Hoogervorst, 2017). The earliest definition of the meaning of work comes from the Job Characteristic Model proposed by Hackman and Oldham (1976). In this model, the meaning of work is composed of three dimensions: skill variety, job identity, and assignment significance. However, scholars believe that the characteristics of work cannot fully represent the meaning of work, which can be relevant to the employees’ belief that their work is meaningful and worthwhile. When an individual feels that an actual bond to work allows them to transcend oneself, that individual forms a sense of work meaning (Bailey and Madden, 2016). The meaning of work could be explored from a multilevel dimension, in spite of no agreement on the intention. In previous research, the meaning of work implies the “optimistic, subjective, and personal experience” derived from the job (Bailey et al., 2018).

The structural division of meaning of work has also been developed from single to multiple dimensions. Early research has proposed three dimensions of work meaning, which include self-perception, work itself, and a sense of balance (Chalofsky, 2003). Studies have also pointed out that the meaning of work consists of three categories. The first is the psychological meaning of work, based on the individual’s judgment on whether the work is meaningful and valuable. The second part is the creation of meaning in work. The meaning of life comes from work, and the individual can realize self-growth by strengthening the perception of work and the world. The third aspect is to advocate friendly relationships and emphasize the wider positive impact of work on others, communities, and society (Steger et al., 2013). Many studies have noticed the dynamic balance of the meaning of work. Lips-Wiersma and Wright mentioned that the meaning of work reflected a sense of balance between two dimensions. The first is the “existence” and “being,” and the second is the “self” and “others.” Specifically, the study believes that the meaning of work comes from two key dimensions. One is the subject of the work, which is self and others. The second is the type of individual needs, which are action needs and existential needs. Action needs and existential needs refer to gaining a sense of meaning by paying attention to different aspects. Action needs are gaining a sense of meaning through the feeling of one’s contribution to the external environment. The existential needs are gaining a sense of meaning through the feeling of self-growth or development. With the research advancement, many techniques are devised to divide the sense of work meaning. Some scholars summarized the dimensions of the sense of work meaning and proposed a model structure to integrate all dimensions. However, no consensus has been reached regarding the sense of work meaning. Lips-Wiersma and Wright proposed the seven-dimensional structure in 2012, including uniting with others and expressing full use of their potential.

Employee’s innovative behavior is a process in which employees generate novel and potentially economic or socially valuable ideas based on their knowledge and experience, which include the production of new products or services through innovative labor or innovative ideas from manufacturing methods and business management thinking. In upgrading public service demand and adaptability of the organization, service quality improvement and client fulfillment can be obtained through employees’ innovative behavior (Amabile, 1988). Extensive research shows that innovative behavior comprises ideas, thinking, and a process that ultimately applies to creative ideas and practice. In employee innovation behavior, employees have creative ideas about problems or propose an innovative solution, by actively putting ideas into specific work practices. The process includes the stage of generating innovative ideas and includes the performance of putting the innovative ideas into practice and behavior (Yuandong and Jisheng, 2011). Thus, employee innovation behavior is the active process that employees use to change existing problems and situations based on their knowledge and a favorable innovation environment. The strategies assist in generating new ideas to develop novel products, services, manufacturing methods, and management, and put them into practice (Kang et al., 2015).

In previous studies, the meaning of work is a dimension of psychological empowerment (Spreitzer, 2007). Individual psychological empowerment can promote creative outcomes in an organization (Roth et al., 2020). Based on the job demands-resources model, psychological empowerment in an organization is a work resource, referred to as the “positive factors” in the work environment. With the natural motivation characteristics, the meaning of work can stimulate employees’ motivation, increase work engagement, and offer a positive impact. Amabile proposed the motivational synergy model pointing out that people would generate creative ideas if they were more committed to work from the heart and not distracted by external factors when solving work problems.

The meaning of work is an essential element to improve the individual’s innovation level, professional skills, creativity-related skills, and motivation following Amabile’s model of creativity and innovation (Amabile and Pillemer, 2012). The meaning of work enhances the individual level of innovation in four ways: first is meaningful work to enhance the individual’s internal motivation. The second is to strengthen the virtuous circle of the creative process. Individuals will actively repeat the creative process whether the attempted result is a success or failure. Increasing the persistence of creativity through this behavior also enhances innovation. The third is that the meaning of work can adjust the relationship between the organization leader’s statement of the innovation task and the individual’s internal innovation motivation. The fourth is to enhance creativity through individual “work orientation.” Members of the organization can find the valuable parts of their work through the sense of work meaning, thereby affecting individual innovation. Some studies revealed the positive effects of job meaning on job performance (Kosfeld et al., 2017) and job motivation (Lips-Wiersma and Wright, 2012). Thus, we proposed the following hypothesis:

*H1*: The meaning of work has a positive impact on the innovative behavior of scientific and technological workers.

### Mediating effect of achievement motivation

David McClelland put forward the Achievement Motivation Theory by researching human needs and motivation. McClelland suggests Three Needs Theory, which proposes that individuals in a working environment should possess three key needs or motivations, which include the need for power, the need for affiliation, and the need for achievement. According to a large number of relevant studies, McClelland found that individuals with higher needs for achievement were more inclined to work independently, get positive feedback from colleagues or leaders, and face certain risks. In such an environment, they are often encouraged to have a better chance of success. This further illustrates that achievement needs can positively improve personal work achievement (Lin et al., 2019). American psychologist Atkinson developed the Achievement Motivation Theory, pointing out that achievement motivation was critical for individuals to achieve their goals. Achieving individual success is the same as avoiding failure. The two conflicting emotions that shape the achievement motivation are the desire for success and the fear of failure. Therefore, the individual achievement motivation is composed of the motivation to succeed and the motivation to avoid failure.

Existing research about achievement motivation is concentrated in the fields of education and business management, as the fields focus on the achievement motivation of students and employees, such as emotional and performance aspects. The achievement motivation is reflected in the employees’ career choices, job performance, and willingness to flow in the leadership cadre of the company management. McClelland points out that people with a high level of achievement motivation and who offer challenging work have the courage to make decisions at work. However, people with low-level achievement motivation and people who offer work with low risk make fewer decisions. The previous research showed that achievement motivation plays an important role in the employee group (Lin et al., 2019). Employees with higher achievement motivation pursue higher performance and prefer to offer skillful and challenging tasks (Eisenberger et al., 2005).

Prior studies have shown that the characteristics of achievement motivation include overcoming and achieving something difficult. The achievement goal theory (Dweck and Leggett, 1988) showed that people with a high level of achievement motivation preferred seeking the challenge during the work process to build the mastery goal orientation and performance goal orientation. These people have the skills to offer better innovative performance when facing more challenges. Moreover, people with a low level of achievement motivation tend to avoid challenges and failures (Durik and Harackiewicz, 2003). Recent research finds that when individuals desire to match the high level of creativity, they will have a higher intrinsic motivation to promote innovative behavior (Li et al., 2020). Therefore, science and technology workers with a high level of achievement motivation spontaneously devote themselves to work to actively discover the work problems and make beneficial attempts. Thus, the process will promote the formation and development of innovative behaviors. As expounded above, the following hypothesis is proposed:

*H2*: The achievement motivation has a positive impact on the innovative behavior of scientific and technological workers.

The meaning of work directly leads to positive work attitudes and behaviors based on a positive emotional experience and motivational state. Many studies used job characteristic model theory and motivation theory to explore different work perspectives. Employees can better obtain physical and mental health and self-realization when they have a deeper understanding of the meaning of work. As a part of psychological empowerment, the meaning of work has a positive impact on intrinsic motivation and satisfaction (Spreitzer, 2007). Early studies validated the transfer effect of the meaning of work on individual positive work motivation and output (Johns, 1992). The previous research also points out that workers with a high level of achievement motivation have a positive mission and offer more meaning in their work. Moreover, they are more inclined to choose challenging tasks and invest in these tasks to gain a sense of accomplishment and satisfaction (Schoen, 2015). Some studies showed that when people pay attention to experience, they will be more motivated to pursue goals (Sedikides et al., 2017) and complete challenges more confidently (Abeyta et al., 2015). As expounded above, the following hypothesis is proposed:

*H3:* The meaning of work has a positive impact on the achievement motivation of scientific and technological workers.

### Moderating effect of organizational innovation climate

The innovative climate is a set of characteristics perceived and measured by the members of organizations and influences the motivation, attitude, belief, and innovative behavior of the members of organizations (Amabile and Pratt, 2016). The innovative climate is the perception of the degree of innovation support for organizational members in their working environment (Tu, 2015). The innovative climate will continue to affect the innovation motivation, innovation attitude, and innovation-related skills of the members of the organization; it will also affect the innovative ability and innovative performance of the organization (Amabile and Pratt, 2016; Gundry et al., 2016). The organizational innovative climate is multi-faceted, and its structure influences the cognition of a single event or organizational phenomenon. Amabile developed the KEYS scale to assess the climate for creativity and innovative climate. It is believed that the innovative climate comprises 10 dimensions, such as incentive mechanism, team support, and autonomy. With an increase in research on innovative climate, many scholars have proposed different dimensions of innovative climate.

At the same time, organizational climate is closely related to organizational culture and social culture. Therefore, Chinese scholars develop a scale of organizational innovative climate suitable for the Chinese environment, considering the similarities and differences between Chinese and Western cultures. For example, Shi compiled the “Innovative Culture Questionnaire of the Chinese Academy of Sciences.” Some studies divide innovation climate into five dimensions based on the Chinese private organizations: team operation, organizational support, leadership effectiveness, work autonomy, and learning growth. Studies also found that the organizational innovative climate is pivotal to promoting employee innovative behavior.

The components of organizational creativity and innovation show that working environment factors will affect creativity based on individual factors. In addition, individual or team creative output is a source of organizational innovation. According to Triadic Reciprocal Determinism Theory, three factors will form a ternary interactive decision system capable of affecting human activities. These factors are individual characteristics and cognition, individual behavior, and the external environment. The Social Cognition Theory explores the relationship between the external environment, individual psychological cognition, and individual behavior, which provides a theoretical basis for explaining how organizational innovation climate moderates the connection between innovation behavior and achievement motivation.

Previous studies show that organizational innovative climates significantly promote organizational citizenship behaviors, such as employee knowledge-sharing behavior (Edú-Valsania et al., 2016), employees’ creative behavior (Jaiswal and Dhar, 2015), and innovation outcomes in the organization (Suliman, 2001). A study found that the team’s innovative climate promotes creative behavior by enhancing individual proactive and adventurous attitudes (Magni et al., 2018). Another study finds that it is critical to improving employee creativity through the interaction between employee motivation and high goal orientation in the organizational environment (Lee and Yang, 2015). To implement the moderating effect of innovation climate, many studies demonstrated the influence of innovation climate on various causal variables. The organizational innovation climate positively moderates the relationship between the voice behavior of ethical leadership and personal creativity (Chen and Hou, 2016). Many studies used the job demands-resources theory to confirm that a high level of work resources can significantly improve the outcome variables and employees’ work performance in a high level of work demanding environment. Innovation is a high work requirement that requires scientific and technological workers to devote themselves to work more fully and use a large number of work resources available to complete their work goals (Alexander and Knippenberg, 2014). Therefore, this research proposes the following hypotheses:

*H4*: Organizational innovative climate positively moderates the relationship between achievement motivation and innovative behavior of scientific and technological workers. Under the high-level organizational innovative climate, the achievement motivation of scientific and technological workers will have a stronger positive impact on innovative behavior.

**FIGURE 1:**
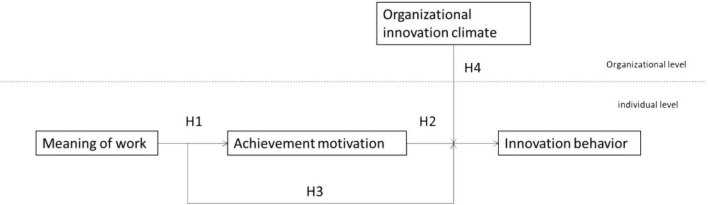
Theoretical model of the study.

After analyzing the previous literature, relevant hypotheses are put forward for the corresponding variables based on a theoretical framework. Figure 1 shows a conceptual model of four variables: meaning of work, achievement motivation, innovative behavior, and organizational innovative climate.

## Materials and methods

### Sample and data

This survey was conducted in Tianjin, China. The participants were scientific and technological workers from universities, research institutes, government departments, and R&D institutions who worked in different industries, such as electronic information, environmental protection, and aerospace. The participants completed the survey from August to December 2020 and filled out the online survey. After a description and an explanation of the study, the process yielded 4,666 valid responses.

Of the approximately 4,666 participants, 3,201 (53.9%) were men, and a considerable percentage of participants were under 40 years old (78.1%). Since the respondents are scientific and technological workers, 2,979 people (63.8%) have a bachelor’s degree or above. Table 1 presents the detailed demographic information of the participants.

**TABLE 1:** Summary of demographics of participants.

Characteristic		*F*	%
Gender	Male	2516	53.9%
	Female	2150	46.1%
Age	<31	1442	30.9%
	31–40	2203	47.2%
	>40	1021	21.9%
Educational attainment	Junior college	929	20%
	Undergraduate	2637	56.5%
	Master’s degree and higher education	1100	23.5%
Major and work relevance	Irrelevant	718	15.4%
	Relevant	3948	84.6%

### Measures

#### Meaning of work

The seven items developed by Lips-Wiersma and Wright (2012) were used to measure the meaning of work in this study. Sample items include “I have a certain social responsibility” and “I experience a sense of belonging.” A five-point scale ranging from 1 (strongly disagree) to 5 (strongly agree) was used to measure the questionnaire items.

#### Achievement motivation

The four items for achievement motivation were adapted from the Achievement Motivation Scale. Sample items include “I prefer different and difficult tasks and take risks.” A five-point scale was applied.

#### Innovative behavior

This variable was measured using five items derived from the two-dimensional aspect of Susanne and Bruce (1994) and translated by Zhu (2009). Sample items include “In my work, I take the initiative to discover and apply new technologies or processes.” A five-point scale was applied.

#### Organizational innovative climate

The scale including five items derived from Wenqin et al. (2010) was used to measure this variable. Sample items include “satisfaction degree of the unit’s science and technology reward system” and “satisfaction with the construction of the unit’s scientific research team.” A five-point scale was applied.

#### Control variables

This study selects age, education level, gender, work, and the highest level of education as the control variables.

### Empirical analysis

#### Common methods variance and multicollinearity

We solve the problem of common method variance from the data collected through the questionnaire (Podsakoff et al., 2012). We tested common methods variance. The result shows that no single factor can explain most of the variation because all scores are below 40%. Therefore, the data we collected are appropriate for the following analysis.

The variance inflation factor (VIF) is used to check the multicollinearity score. If the VIF score is lower than 4.00, the data do not have multicollinearity problems. After we analyzed the data, it turned out that no multicollinearity was found in the data because all VIF scores were below 4.

#### Reliability and validity

SmartPLS software was employed to verify the reliability and validity of the variables. First, Cronbach’s alpha index was used to test the reliability of each variable. All variables were reliable and acceptable for use in this study. In addition, the test outcomes were displayed in Table 2, revealing that all Cronbach’s alphas were greater than 0.8, showing that the correlation between different items in the scale was relatively high (Cronbach, 1951).

**TABLE 2:** SEM measurement model analysis results.

	AVE	Composite reliability	Cronbach’s alpha	rho_A	Q^2^
MW	0.661	0.931	0.913	0.917	0.545
AM	0.627	0.870	0.808	0.832	0.377
IB	0.759	0.940	0.921	0.923	0.630
OIC	0.771	0.944	0.927	0.948	0.645

MW, meaning of work; AM, achievement motivation; IB, innovation behavior; OIC, organizational innovation climate.

The evaluation indicators of convergent validity include the reliability coefficient (CR) of variables and the average variance extracted (AVE) of potential variables. The CR of the potential variable measures the degree of agreement between the observed variables of the same potential variable. A higher CR value indicates a higher degree of correlation among observed variables. Table 2 presents the test results, which reveal that the CR of all variables is greater than 0.8, ensuring the validity of the scale and questionnaire data (Bagozzi, 1981). The AVE expresses a total mean-variance of the observed variable, which is explained by the potential variable relative to the measurement error. A value greater than 0.5 is the observed variable that can effectively reflect the corresponding potential variable (Bagozzi, 1981). Table 2 showed the results, revealing that the AVE is always greater than 0.6 and showing that the convergent validity is good.

To test the divergent validity of variables, this study used the method proposed by Fornell and Larcker (1981) to compare the square root of the average variance extracted (AVE) with the correlation coefficient between the potential variables (Fornell and Larcker, 1981). If the square root of each AVE of a potential variable is greater than the correlation coefficient between potential variables, it indicates that the correlation between measurement items of different potential variables is low, and the divergent validity of the scale meets the analysis requirements. The test results are shown in Table 3. The bold words on the diagonal show the square root value of each AVE of the potential variable. It can be found that the square root value of each AVE of the potential variable is greater than the correlation coefficient between it and the other potential variables, which indicates the study has good divergent validity.

**TABLE 3:** Mean, standard deviation, and correlation values of study variables.

	1	2	3	4	5	6	7	8
Age								
Gender	0.024							
Educational attainment	0.048	–0.028						
Major and work relevance	–0.090	0.062	0.255					
Meaning of work	–0.051	0.032	0.121	0.282	**0.813**			
Achievement motivation	–0.150	–0.022	0.102	0.194	0.363	**0.792**		
Innovation behavior	–0.084	0.030	0.173	0.239	0.471	0.664	**0.871**	
Organizational innovation climate	–0.011	–0.026	0.055	0.175	0.811	0.273	0.319	**0.878**
*M*	1.461	1.968	3.052	3.091	3.508	3.252	3.685	3.388
*SD*	0.499	0.843	0.872	1.298	0.635	0.681	0.697	0.712

AVE, average variance extracted. The square root of the AVE is on the diagonal in bold.

## Result

**FIGURE 2:**
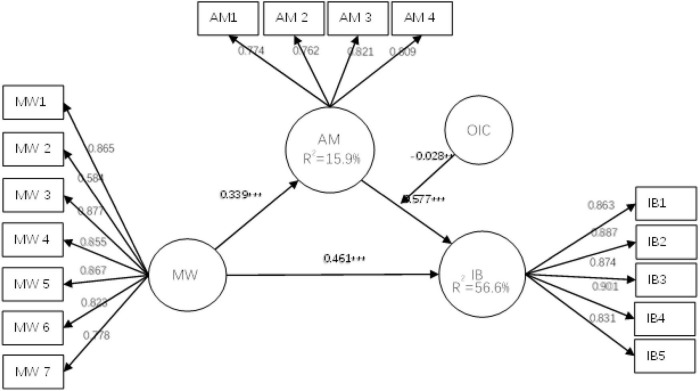
Final empirical research model.

In this study, we tested the hypothesis in SmartPLS. First, we estimated each path parameter of the model through SmartPLS. Second, bootstrapping with 5,000 bootstrap samples was applied to test the significance of the path coefficients between variables and to generate 95% confidence intervals. The results are presented in Table 4. According to the model in this study, all variables and the loading factor are shown in Figure 2.

**TABLE 4:** Standardized regression weights for direct and indirect paths with hypotheses results.

Hypotheses	Paths	Path coefficient	*SD*	*t*-statistic	*P*-value	Results
H1	MW→IB	0.461	0.022	20.542	0.000	Supported
H2	AM→IB	0.577	0.012	48.309	0.000	Supported
H3	MW→AM	0.339	0.015	27.475	0.000	Supported
H4	AM*OIC→IB	-0.028	0.009	3.080	0.002	Not supported

MW, meaning of work; AM, achievement motivation; IB, innovation behavior; OIC, organizational innovation climate.

### Mediating effect test

A great fit was shown in the model to proceed to the next step and test the hypothesis. First, H1 predicted that the meaning of work positively influenced the innovation behavior of science and technology workers. Table 4 reported the result (β = 0.461, *p* < 0.001), revealing that the meaning of work positively influences innovation behavior. Moreover, H2 predicted that the achievement motivation would positively improve the innovation behavior of science and technology workers. The regression coefficient between achievement motivation and innovation behavior, shown in Table 4, is statistically significant (β = 0.577, *p* < 0.001), as proved by H2. Moreover, H3 predicted that the meaning of work positively affects the achievement motivation of science and technology workers. The meaning of work plays a vital role in achievement motivation (β = 0.339, *p* < 0.001), which supports H3. The meaning of work indirectly impacts innovative behavior and is mediated by achievement motivation.

### Moderating effect test

**FIGURE 3:**
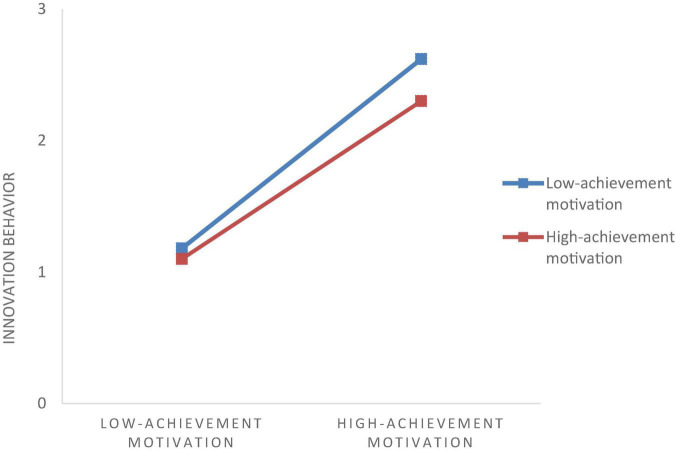
Moderating effect of organizational innovation climate between achievement and innovation behavior.

Table 4 reports the results, showing that organizational climate is a moderator variable. We test the moderating effect on achievement motivation and innovation behavior. Regression coefficients reported statistically significant results for interaction terms and innovative behavior (γ = -0.028, *p* < 0.01), which failed to support H4. Figure 3 intuitively reflected that as the innovation behavior increased with achievement motivation, organizational innovation climate, as a moderator variable, played a significant moderating role. Figure 3 shows that the lower the organizational innovation climate perceived by employees, the stronger the role of achievement motivation in promoting innovative work behavior, which is not supported by H4.

### Importance-performance map analysis of meaning of work

The above-mentioned study has proved that the meaning of work positively improves innovation behavior directly and indirectly. Thus, we use an IPMA for further analysis of the meaning of the work of scientific and technological workers and identify specific factors that make innovative behavior relatively high. However, the score is relatively poor. The performance map analysis is vital for practical application. The step is to set a dimension according to the average level of the potential variable and extend the standard results, showing path coefficient evaluation (Ringle and Sarstedt, 2016).

**FIGURE 4:**
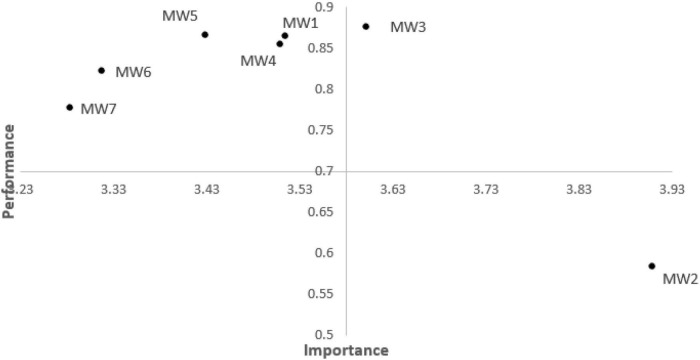
Importance-performance map for meaning of work indicators. MW, meaning of work.

As shown in Table 5 and Figure 4, the importance of MW3 (sense of calling) has a high priority, and performance is slightly above average. Compared with MW3, the MW4 indicators (full play to professional expertise), MW5 (sense of self-achievement), and MW1(sense of belonging) have the highest priority for performance improvement. However, the performance of MW4, MW5, and MW1 is slightly below average. The importance of MW6 (personal development space) and WM7 (career development needs) are also above average, and their performance is far below average. Moreover, MW2 (sense of social responsibility) has a lower priority for innovative behavior promotion owing to the lower importance and higher performance.

**TABLE 5:** Importance and performance data for study indicators and constructs.

Constructs	Codes	Indicators	Indicator important	Indicator performance
Meaning of work	MW1	Sense of belonging	0.87	3.52
	MW2	Sense of social responsibility	0.58	3.91
	MW3	Sense of calling	0.88	3.60
	MW4	Full play to professional expertise	0.86	3.51
	MW5	Sense of self-achievement	0.87	3.43
	MW6	Personal development space	0.82	3.32
	MW7	Career development needs	0.78	3.28

## Discussion

For scientific and technological workers, innovative behavior is important for work performance. Previous studies indicated that many factors affect the innovative behavior of science and technology workers, which include individual factors, group factors, and organizational factors. We explore the meaning of work, which is the deeper motivation factor that affects scientific and technological workers. Furthermore, we explore the influence of the meaning of the work of scientific and technological workers on innovative behavior. We draw the following major conclusions:

First, the meaning of work positively improves the innovative work behavior of scientific and technological workers, which is consistent with previous studies. Previous studies have supported the finding that people who think their work is meaningful may personally invest in their work (May et al., 2004). Also, according to the dynamic componential model of creativity and innovation, it is proposed that the meaning of work can be improved in four ways to increase the level of innovation of individuals (Amabile and Pratt, 2016). Through empirical research on Chinese scientific and technological workers, this research proves that the more scientific and technological workers realize the meaning of work, the more they realize that their work is beneficial to life and society, which is the embodiment of self-worth. This study provides empirical support for the dynamic componential model of creativity and innovation in the context of Chinese scientific and technological workers. Meaningful work experiences, including personal perceptions of greater benefit (Steger et al., 2013), may increase employees’ willingness to use their abilities and energy to achieve innovative behaviors (Kashdan et al., 2004). The scientific and technological workers will achieve more lasting inner motivation to innovate through meaningful work, and they can acquire more positive experiences from the innovative process.

Second, the result shows that the meaning of work indirectly impacts innovative behavior, and it is partially mediated by achievement motivation. The meaning of the work of scientific and technological workers can directly promote their innovative behaviors through achievement motivation. Existing research shows that the meaning of work, as part of psychological empowerment, has a positive effect on intrinsic motivation and satisfaction (Spreitzer, 2007). In addition, a meaningful sense of work indicates that employees are intrinsically motivated to work (Steger et al., 2013; Amabile and Pratt, 2016) because they find purpose, value, and meaning in their tasks. Because of their intrinsic motivation, employees may be inclined to translate their motivation into higher-level efforts (e.g., generating, promoting, and realizing their innovative activities) aimed at benefiting the organization in its (innovative) achievements (Fuller and Hester, 2010; Yuan and Woodman, 2010; Yidong and Xinxin, 2013). We extend the motivational perspective that has been prominent in creativity and innovation research (Liu et al., 2016). That is, the meaning of work can be linked to employee innovative behavior through achievement motivation (Amabile and Pratt, 2016). This shows that the meaning of the work of scientific and technological workers is a key factor in inspiring achievement motivation. Scientific and technological workers who embrace the meaning of work will increase their work involvement, since their work engagement is important and valuable. The meaning of work can also stimulate their sense of accomplishment after completing their goals.

Third, the organizational innovative climate has a negative moderating effect on the achievement motivation and innovative behavior of scientific and technological workers. This is different from previous studies, which have shown that organizational innovation climate significantly promotes organizational citizenship behaviors, such as employee knowledge-sharing behavior (Edú-Valsania et al., 2016), employee creative behavior (Jaiswal and Dhar, 2015), and innovation outcomes in organizations (Suliman, 2001). Some studies believe that a high level of organizational innovation climate improves employees’ self-efficacy, motivates employees to make more innovative ideas and behaviors, and improves the utilization of knowledge and other resources (Yuandong and Jisheng, 2011).

The achievement motivation of scientific and technological workers has a strong positive influence on innovative behavior when the organizational innovation climate is low. Leaders do not pay enough attention to innovation, and the resources related to innovation are relatively difficult to obtain when the organizational innovation climate is low. This challenging environment will improve their innovative behavior because stimulating scientific and technological workers can solve problems more actively. Thus, the effect of achievement motivation on innovative behavior is more obvious. Individuals with higher needs for achievement are more likely to work independently, get positive feedback from colleagues or leaders, and face certain risks. Specifically, employees with high achievement motivation not only tend to take on more challenging, high-goal jobs but also emphasize value pursuit. In such an environment, they are often encouraged to have a better chance of success (Schoen, 2015). In addition, research has shown that employees with high achievement motivation may have some negative attitudes in a climate that encourages innovation, including dissatisfaction with colleagues and subjectivity. For employees, this strong environment produces negative emotions, such as great stress (Janssen, 2011). This study also confirmed that in the context of a strong level of organizational innovative climate, the achievement motivation as a context-related trait may be reduced, which weakens the positive effect of the achievement motivation of scientific and technological workers on innovative behavior.

### Theoretical contributions

Through empirical research, we verified and expanded the theory of creativity and innovative components by conducting the effect of the meaning of work on innovation behavior (Amabile and Pratt, 2016). Fewer empirical studies demonstrate the meaning of work and its effect on innovative behavior. This article focuses on the meaning of work as a factor that stimulates the deeper motivation of scientific and technological workers. The empirical research confirms that the meaning of work directly enhances innovative behaviors of scientific and technological workers and stimulates their vitality and investment.

However, the conclusions in our research are not consistent with previous studies. Many studies have shown that the organizational innovative climate positively improves the performance of individuals in the different organizational processes (Edú-Valsania et al., 2016). Unlike previous studies, this study demonstrates that the organizational innovative climate negatively affects the achievement motivation of scientific and technological workers and their innovative behavior.

We consider two possible reasons. Trait activation theory makes each individual react strongly, conceals the individual’s potential traits, and erases individual differences. Achievement motivation, which is an individual trait to the situation, is more likely to be activated and play an important role when the organizational innovation climate is weaker and the work environment is more challenging. As the previous research has mentioned, trait changes need to be motivated based on weak to moderate situational information related to the trait (Tett and Guterman, 2000). Another reason is that people with high achievement motivation usually desire success. However, the innovative process is unpredictable, controversial, and may conflict with other job requirements. Therefore, employees with high achievement motivation may develop some negative attitudes when they are in an atmosphere that encourages innovation. The results include dissatisfaction with colleagues and subjectivity. For employees, this strong environment can produce bad emotions, such as enormous pressure (Janssen, 2011). Moreover, small groups in an organization are prone to appear in a higher-level organizational innovative climate due to competition for innovative resources. This may lead to greater conflicts and ultimately negatively affect the performance of the individual and the organization (Newman et al., 2020).

This article enhances a greater understanding of the different mechanisms of climate by emphasizing the influence of achievement motivation of scientific and technological workers on innovative behaviors in different organizational contexts. This lays a certain foundation for future study.

### Practical implications

This article focuses on the innovative behavior of scientific and technological workers and provides a different perspective on how to improve innovative behavior. Our findings offer some practical implications.

In contemporary society, promoting the innovative behavior of employees, especially the innovative behavior of science and technology workers, gives incentives to improve the innovative extrinsic motivation of managers and awaken employees’ self-consciousness. This article reveals that the meaning of work is rarely valued in organizations. However, it can provide scientific and technological workers with deeper intrinsic motivation and enhance their innovative behavior. This is consistent with past findings that people who pursue meaning tend to have stronger intrinsic motivations (Fiorito et al., 2020).

From the findings, organizations should provide external incentives, such as material rewards, and pay attention to the pursuit of the meaning of work. Specifically, it is important to improve the sense of belonging and the sense of mission of scientific and technological workers in the organization.

Moreover, we provide some practical discoveries through the IPMA. Important is a sense of belonging, calling, self-achievement, and the extent to play the professional expertise of scientific and technological workers on the meaning of work. Thus, managers can prioritize the four aspects of the meaning of work to improve the innovative behavior of scientific and technological workers, as these indicators offer a higher (above average) importance. Considering the sense of belonging, self-achievement, and extent to play the professional expertise offer a low performance for scientific and technological workers. These three indicators must be improved.

Managers must create an environment where scientific and technological workers can feel more belonging. A high sense of belonging enhances good relationships with colleagues in the work environment and enhances the individual’s interpersonal relationships and social identity.

Second, this paper verifies the mediating effect of achievement motivation for meaningful work and innovative behavior. Organizations should attach more importance to the level of achievement motivation of scientific and technological workers. The level of achievement motivation can be used as a reference for different job assignments when the organization conducts recruitment activities. Science and technology workers with high levels of achievement motivation are more competent for positions that require higher requirements and innovative behaviors. Moreover, training is vital for scientific and technological workers to undertake tasks that require certain challenges and high goals, which can help them improve their self-development.

Third, the results are different from that of past studies regarding the creation of an organizational innovation climate, which show the stronger the organizational climate, the better the innovative behavior. An excessively strong organizational innovation atmosphere will inhibit the expression and influence of some innovation-related characteristics. For scientific and technological workers with a low level of achievement motivation, the leader can provide them with a strong organizational innovation climate, such as allowing them to have strong work autonomy. However, a high level of achievement motivation of scientists enhances an overly strong organizational innovative climate, which may erase the expression of achievement motivation. We suggest that the appropriate organizational innovation climate will stimulate the scientific and technological workers and promote their innovative behavior.

### Limitations and directions for future research

First, the data collected are cross-sectional, which has certain limitations in explaining the causal relationship between variables. In future studies, longitudinal data can be used. Also, future research may consider not only surveying scientific and technological workers themselves but also surveying their colleagues or supervisors to obtain more comprehensive data. Second, this research mainly discusses achievement motivation as a mediator between the meaning of work and innovative behavior. Other mediator variables can be added in future studies like contextual variables. The third is that the research shows that organizational innovation climate is a moderating variable. For certain boundary conditions, specific boundary conditions can be studied and discussed in the follow-up research, and more specific research conclusions can be drawn. Finally, our research was conducted only in a part of China. For this reason, as future prospects, the study can be expanded to other regions and countries.

## Data availability statement

The datasets presented in this article are not readily available because the data are not publicly available due to privacy or ethical restrictions. Requests to access the datasets should be directed to KL, kexin_liang@tju.edu.cn.

## Ethics statement

The studies involving human participants were reviewed and approved by the National Academy of Innovation Strategy of China. The patients/participants provided their written informed consent to participate in this study. Written informed consent was obtained from the individual(s) for the publication of any potentially identifiable images or data included in this article.

## Author contributions

KL contributed to the conception or design of the work. SL and JL provided approval for publication of the content. YZ revised the work critically for important intellectual content. All authors contributed to the article and approved the submitted version.

## Conflict of interest

The authors declare that the research was conducted in the absence of any commercial or financial relationships that could be construed as a potential conflict of interest.

## Publisher’s note

All claims expressed in this article are solely those of the authors and do not necessarily represent those of their affiliated organizations, or those of the publisher, the editors and the reviewers. Any product that may be evaluated in this article, or claim that may be made by its manufacturer, is not guaranteed or endorsed by the publisher.
